# Assessing long-range contributions to the charge asymmetry of ion adsorption at the air–water interface†

**DOI:** 10.1039/d0sc01947j

**Published:** 2020-10-05

**Authors:** Stephen J. Cox, Dayton G. Thorpe, Patrick R. Shaffer, Phillip L. Geissler

**Affiliations:** Department of Chemistry, University of Cambridge Lensfield Road Cambridge CB2 1EW UK; Chemical Sciences Division, Lawrence Berkeley National Laboratory Berkeley CA 94720 USA; Department of Physics, University of California Berkeley CA 94720 USA; Department of Chemistry, University of California Berkeley CA 94720 USA geissler@berkeley.edu

## Abstract

Anions generally associate more favorably with the air–water interface than cations. In addition to solute size and polarizability, the intrinsic structure of the unperturbed interface has been discussed as an important contributor to this bias. Here we assess quantitatively the role that intrinsic charge asymmetry of water's surface plays in ion adsorption, using computer simulations to compare model solutes of various size and charge. In doing so, we also evaluate the degree to which linear response theory for solvent polarization is a reasonable approach for comparing the thermodynamics of bulk and interfacial ion solvation. Consistent with previous works on bulk ion solvation, we find that the average electrostatic potential at the center of a neutral, sub-nanometer solute at the air–water interface depends sensitively on its radius, and that this potential changes quite nonlinearly as the solute's charge is introduced. The nonlinear response closely resembles that of the bulk. As a result, the net nonlinearity of ion adsorption is weaker than in bulk, but still substantial, comparable to the apparent magnitude of macroscopically nonlocal contributions from the undisturbed interface. For the simple-point-charge model of water we study, these results argue distinctly against rationalizing ion adsorption in terms of surface potentials inherent to molecular structure of the liquid's boundary.

## Abstract 2

Cations and anions have different affinities for the air-water interface. The intrinsic orientation of surface molecules suggests such an asymmetry, but the bias is dominated by solvent response that is spatially local and significantly nonlinear.

## Graphical abstract

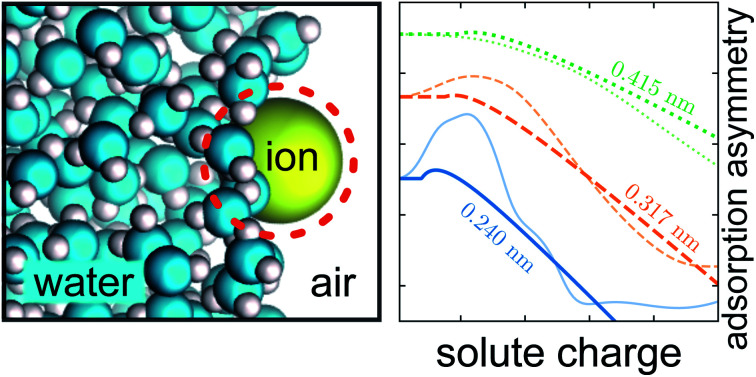

Counter to expectations from conventional theories of solvation, there is a large body of both computational and experimental evidence indicating that small ions can adsorb to the air–water interface.^1–9^ Implications across the biological, atmospheric and physical sciences have inspired efforts to understand the microscopic driving forces for ions associating with hydrophobic interfaces in general.^10–21^ A particular emphasis has been placed on understanding ion specificity, *i.e.*, why some ions exhibit strong interfacial affinity while others do not. Empirical trends indicate that ion size and polarizability are important factors, as could be anticipated from conventional theory. More surprisingly, the *sign* of a solute's charge can effect a significant bias, with anions tending to adsorb more favorably than cations.

Here we examine the microscopic origin of this charge asymmetry in interfacial ion adsorption. We specifically assess whether the thermodynamic preference can be simply and generally understood in terms of long-range biases that are intrinsic to an aqueous system surrounded by vapor. By “long-range” and “nonlocal” we refer to macroscopically large scales, *i.e.*, collective forces that are felt at arbitrarily long distance. Such a macroscopically long-range bias is expected from the air–water interface due to its average polarization, and by some measures the bias is quite strong. By contrast, “local” contributions comprise the entire influence of a solute's microscopic environment, including electrostatic forces from molecules that are many solvation shells away – any influence that decays over a sub-macroscopic length scale.

The importance of macroscopically nonlocal contributions has been discussed extensively in the context of ion solvation in bulk liquid water, which we review in Section 1 as a backdrop for interfacial solvation. The notion that such contributions strongly influence charge asymmetry of solvation at the air–water interface has informed theoretical approaches and inspired criticism of widely used force fields for molecular simulation.^22,23^ A full understanding of their role in interfacial adsorption, however, is lacking.

In the course of this study, we will also evaluate the suitability of dielectric continuum theory (DCT) to describe the adsorption process. DCT has provided an essential conceptual framework for rationalizing water's response to electrostatic perturbations. But a more precise understanding of its applicability is needed, particularly for the construction of more elaborate models (*e.g.*, with heterogeneous polarizability near interfaces^24–26^) and for the application of DCT to evermore complex (*e.g.*, nanoconfined^27,28^) environments.

## 1 Charge asymmetry in bulk liquid water

Our study of *interfacial* charge asymmetry is strongly informed by previous work on the solvation of ions in *bulk* liquid water. In this section we review important perspectives and conclusions from that body of work, as a backdrop for new results concerning ions at the air–water interface.

### 1.1 Distant interfaces and the neutral cavity potential

A difference in adsorption behaviors of anions and cations is foreshadowed by the fact that ion solvation in models of bulk liquid water is also substantially charge asymmetric. Born's classic model for the charging of a solute captures the basic scale of solvation free energies, as well as their rough dependence on a solute's size.^29^ We will characterize the size of a solute by its radius *R* of volume exclusion, the closest distance that a water molecule's oxygen atom can approach without incurring a large energetic penalty. Contrary to Born's result, computer simulations indicate that the sign of the charge of small ions can significantly influence their charging free energy *F*_chg_(*q*, *R*), *i.e.*, the work involved in reversibly introducing the solute's charge *q*.^30–39^ This dependence is most easily scrutinized for simple point charge (SPC) models of molecular interactions, where an ion's charge can be varied independently of its other properties. In SPC/E water,^40^ for instance, charging a solute roughly the size of fluoride (*R*_F_ ≈ 0.317 nm) has an asymmetry, *F*_chg_(*e*, *R*_F_) − *F*_chg_(−*e*, *R*_F_) ≈ 16 kcal mol^−1^, almost 30 times larger than thermal energy *k*_B_*T*. Here, *e* is the magnitude of an electron's charge.

The ultimate origin of charge asymmetry in liquid water is of course the inequivalent distribution of positive and negative charge in a water molecule itself. On average, the spatial distribution of positive and negative charge is uniform in the bulk liquid, but any breaking of translational symmetry will manifest the distinct statistics of their fluctuating arrangements. A neutral, solute-sized cavity in water, for example, experiences an immediate environment in which solvent molecules have a nonvanishing and spatially varying net orientation. The internal charge distributions of these oriented solvent molecules generate a nonzero electric potential at the center of the cavity, whose sign and magnitude are not simple to anticipate. By our characterization, this electrostatic bias is *local* in origin – the total contribution of molecules beyond a distance *r* from the cavity decays to zero as *r* increases.

The inequivalent spatial distribution of positive and negative charge in water can generate spatially *nonlocal* biases as well, effects that extend over arbitrarily large distances. Any point in the bulk liquid is macroscopically removed from the physical boundaries of the liquid phase (*e.g.*, interfaces with a coexisting vapor phase), but those distant boundaries may nonetheless impact the thermodynamics of bulk ion solvation. This expectation stems from a textbook result of electrostatics: an infinitely extended (or completely enclosing) dipolar surface, with polarization pointing along the surface normal, generates a discontinuity in electric potential. This voltage offset does not decay with distance from the interface, and thus meets our criterion for macroscopic nonlocality. A two-dimensional manifold of polarization density is certainly a crude caricature of a liquid–vapor interface, but for a polar solvent whose orientational symmetry is broken at its boundaries, a similarly long-range potential from the interface is expected to bias the solvation of charged solutes, even macroscopically deep inside the liquid phase.

**Fig. 1:**
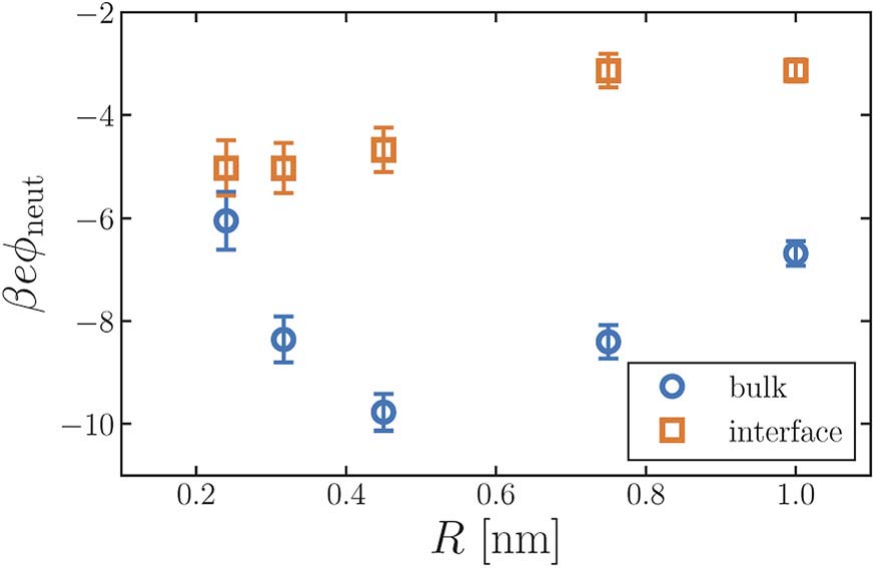
The average electric potential *ϕ*_neut_ at the center of a neutral cavity varies considerably with the cavity's radius *R*. Moreover, this dependence differs for the solute at *z* = *z*_liq_ (“bulk”) and *z* = *z*_int_ (“interface”). The error bars indicate 95% confidence intervals.

The average electric potential *ϕ*_neut_ at the center of a neutral cavity, which we call the “neutral cavity potential”, sums these local and extremely nonlocal contributions. The former depends on the cavity's size (or more generally on the geometry of the solute represented by the cavity). The latter, interfacial contribution should, by contrast, be insensitive to such microscopic details, since the distant surface is unperturbed by the solute. The net electrostatic bias from these two sources can be straightforwardly calculated in computer simulations, not only for SPC models but also with *ab initio* approaches.^14,30,31,41^Fig. 1 shows *ϕ*_neut_(*R*) for cavities in bulk liquid SPC/E water (properly referenced to vapor following ref. 37). Negative potentials of a few hundred mV, varying by nearly a factor of two as *R* grows from 0.2 nm to 1 nm, echo results of previous studies.^42^ Distinguishing quantitatively between local and nonlocal contributions to *ϕ*_neut_, however, is surprisingly confounding, even for the exceedingly strict definition of nonlocality considered here.

One strategy to remove local contributions from *ϕ*_neut_ is to consider the limit *R* = 0. In this extreme case the probe – in effect a neutral, non-volume excluding solute – does not break translational symmetry and induces no structural response. Given the lack of local structure, the presumably nonlocal quantity *ϕ*_neut_(0) = *ϕ*_surf_ is often called the “surface potential”. Lacking volume exclusion, however, this probe explores the liquid phase uniformly, including even the interior of solvent molecules where electrostatic potentials can be very large. A disturbing ambiguity results: the value of *ϕ*_surf_ can be sensitive to modifications of a solvent model that have no impact on the solvation of any volume-excluding solute. Ref. 39 and 41 illustrate this issue vividly, constructing ‘smeared shell’ variants of SPC models with identical solvation properties but very different values of *ϕ*_surf_. This variation in surface potential corresponds to differences in the so-called Bethe potential, which is discussed further in the ESI.†

A related, and somewhat more molecular, approach to isolating the electrostatic bias from a distant phase boundary is to sum contributions to *ϕ*_neut_ only from molecules that reside in the interfacial region. For a macroscopic droplet of liquid water, one could classify each molecule in a given configuration as either interfacial or bulk based on its position relative to the interface. The restricted sum1
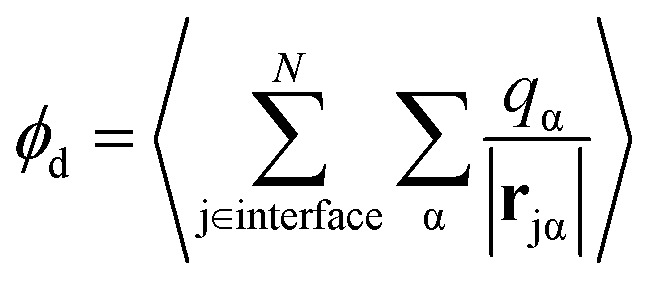
could then be considered as a macroscopically long-ranged, surface-specific component of *ϕ*_surf_ that is appropriately insensitive to a solvent molecule's internal structure. Here **r**_jα_ denotes the position of site α in molecule j, whose charge is *q*_α_, relative to the center of the droplet. *ϕ*_d_ depends significantly, however, on the way molecules are notionally divided between surface and bulk. This dependence, which has been demonstrated previously,^43,44^ we calculate explicitly and generally in the ESI.† Written in the form
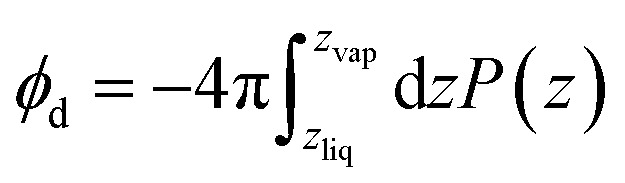
where *P*(*z*) is the solvent dipole density at a displacement *z* from the interface, it reveals *ϕ*_d_ as the well-known “dipole component” of the surface potential.^30,31,39,41,45–47^ Here, *z*_liq_ and *z*_vap_ indicate points within the bulk liquid and bulk vapor, respectively.

For SPC/E water, a surface/bulk classification in eqn (1) based on the position of a water molecule's center of charge gives a value *ϕ*^center^_d_ = −40 mV that differs from an oxygen atom-based classification, *ϕ*^O^_d_ = 240 mV, even in sign.‡‡We define a molecule's center of charge according to the charged sites that specify a particular SPC model. In the case of SPC/E water, this center is displaced from the oxygen atom by approximately 0.029 nm along the molecular dipole. Because water molecules are not point particles, there is no unique way to define an interfacial population, and as a result no unique value of *ϕ*_d_, though attempts have been made to define an optimal choice.^44^ And because molecules near the liquid's boundary are not strongly oriented on average, the range of plausible values for *ϕ*_d_ is as large as their mean.

The ambiguities plaguing interpretations of *ϕ*_surf_ and *ϕ*_d_ are one and the same. Indeed, if we consider an interfacial population of charged sites rather than intact molecules, then *ϕ*_surf_ and *ϕ*_d_ become equal. (When defining an interface of intact molecules, *ϕ*_surf_ and *ϕ*_d_ differ by the so-called Bethe potential, whose analogous ambiguity is described in ESI.†) *ϕ*_neut_ has been characterized as a two-interface quantity,^12,14,41,46–48^ combining the bias *ϕ*_d_ from the distant solvent–vapor interface together with the remaining “cavity” bias *ϕ*_c_ = *ϕ*_neut_ − *ϕ*_d_ from the local solute–solvent interface. From the perspective we have described, these two interfaces are not truly separable, even if a macroscopic amount of isotropic bulk liquid intervenes between them – they must be defined consistently, and the manner of definition substantially influences the change in electrostatic potential at each interface. This is not to say that such a decomposition cannot be useful. Indeed, for computationally demanding *ab initio* approaches it can be convenient to consider local and nonlocal contributions to *ϕ*_neut_ such that, in a first step, *ϕ*_c_ can be obtained from relatively small simulations of the bulk under periodic boundary conditions. The effects of *ϕ*_d_ can then be accounted for in a subsequent step involving simulations of the neat air–water interface. Such an approach was used to good effect in ref. 30 to calculate the solvation free energy of LiF. Nonetheless, this still amounts to an arbitrary choice of dividing surface,^30,39,41^ making it challenging to assign a physical interpretation to *ϕ*_d_ and *ϕ*_c_ individually. Different, and equally plausible, ways of partitioning molecules can give different impressions of the two interfaces. Only the sum *ϕ*_neut_ = *ϕ*_c_ + *ϕ*_d_ is unambiguous.

Establishing an absolute electrostatic bias on the bulk liquid environment due to a distant interface is thus highly problematic for water. A direct scrutiny of this nonlocal contribution, based on the fundamentally ambiguous potential *ϕ*_d_, is untenable. Instead, we assess the relative importance of local and nonlocal biases by comparing the solvation properties of different ions. Local contributions can depend sensitively on features like solute size *R* and charge *q*, while macroscopically nonlocal contributions cannot. Long-range influence of the interface might therefore be clarified by dependence of the neutral cavity potential on *R*. In particular, dominance by the distant liquid–vapor interface would imply weak variation of *ϕ*_neut_ with solute size, which influences only microscopically local structure. The solute size-dependence shown in Fig. 1 does not support such a dominance. Growing the cavity from *R* = 0.24 nm to 0.5 nm lowers *ϕ*_neut_ by roughly 100 mV, followed by an increasing trend for larger cavities. As emphasized in ref. 30 and 41, the role of local charge asymmetry is far from negligible over this range of solute size.

It is tempting to expect the large-*R* behavior of *ϕ*_neut_ to reveal a strictly interfacial component, since local forces attenuate in magnitude when solvent molecules cannot approach the probe position closely. As others have noted,^41,42^ however, neutral cavities larger than *R* = 1 nm induce a solvent environment with the basic character of the air–water interface.^49^ In the limit of large *R*, drying at the solute–solvent interface will generate a cavity potential that cancels the oppositely oriented distant interface with the vapor phase, yielding *ϕ*_neut_ ≈ 0.§§While the vapor phase is very dilute at ambient temperature, its nonzero density does yield an average potential different from the vacuum environment of a volume-excluding cavity. Here we neglect this small distinction. This asymptotic cancellation should begin for nanoscale cavities, though effects of local interface curvature may cause *ϕ*_neut_ to decay slowly towards zero. Judging from our results, there is no intermediate plateau value of *ϕ*_neut_ that could reasonably be assigned to a single liquid–vapor interface.

### 1.2 Solvation thermodynamics and the asymmetry potential

The difficulty of uniquely identifying a surface dipole component of *ϕ*_neut_ notwithstanding, the relevance of such neutral probe quantities for ion solvation thermodynamics has also been thoroughly examined.^10,14,30–32,34,35,39,46,47,50–53^ As an essential thermodynamic measure of solvation, we examine the free energy change *F*_solv_(*q*, *R*) when a solute ion is removed from dilute vapor and added to the liquid phase. This change could be evaluated along any reversible path that transfers the solute between phases, and different paths can highlight different aspects of solvent response. For studying charge asymmetry, a particularly appealing path first creates a neutral, solute-sized cavity in the liquid, with reversible work *F*_cav_(*R*). The second step, whose free energy change *F*_chg_(*q*, *R*) was discussed above, introduces the solute's charge.^2^ The charge asymmetry of interest compares solvating a cation and anion of the same size; since *F*_cav_ is insensitive to the solute's charge, its contribution to *F*_solv_ = *F*_cav_ + *F*_chg_ cancels in the difference

2 *F*_solv_(*q*,*R*) − *F*_solv_(−*q*,*R*) = *F*_chg_(*q*,*R*) − *F*_chg_(−*q*,*R*)

3 ≡2*qψ*(*q*, *R*)

Eqn (3) defines an *asymmetry potential ψ*, an analogue of *ϕ*_neut_ that accounts for solvent response.

The connection between *ψ*(*q*, *R*) and *ϕ*_neut_ can be made precise through a cumulant expansion of *F*_chg_ in powers of *q*,^10,14,34,35,54^4
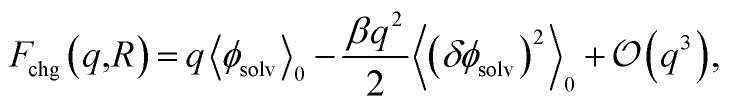
where 〈⋯〉_0_ denotes a canonical average in the presence of a neutral solute-sized cavity, *ϕ*_solv_ is the fluctuating electric potential at the center of the cavity due to the surrounding solvent (so that *ϕ*_neut_ = 〈*ϕ*_solv_〉_0_), and *δϕ*_solv_ = *ϕ*_solv_ − *ϕ*_neut_. The 
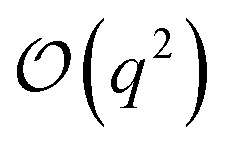
 term in eqn (4) describes linear response of the solvent potential *ϕ*_solv_ to the solute's charging. This response, which could be captured by a Gaussian field theory à la DCT, is charge symmetric by construction. The asymmetry potential 
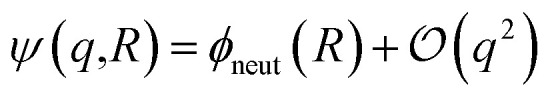
 is therefore equivalent to *ϕ*_neut_ within linear response.

**Fig. 2:**
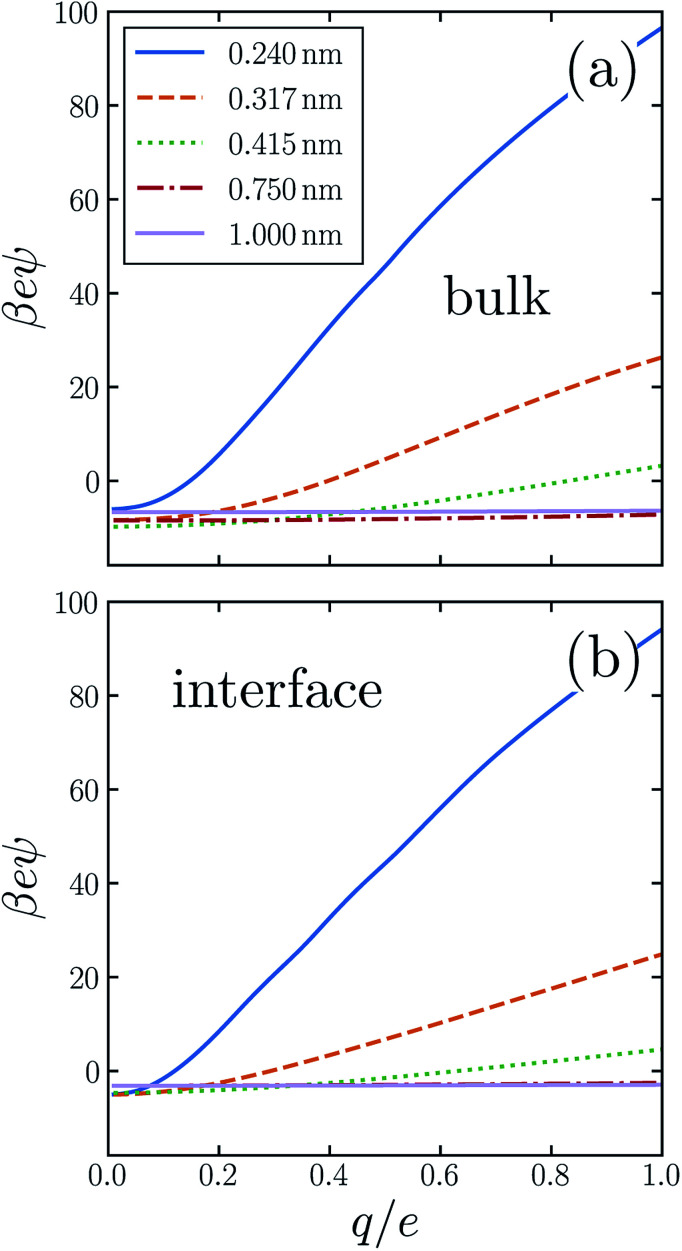
Ion solvation in water is both asymmetric and non-linear, as quantified by the asymmetry potential *ψ*(*q*, *R*; *z*). Results are shown for solutes (a) in the bulk liquid, and (b) near the air–water interface, spanning ranges of charge 0 < *q* ≤ *e* and solute size 0.24 ≤ *R* ≤ 1.0 nm (see legend). Both in the bulk and at the interface, *ψ* < 0 for small *q*, indicating that weakly charged cations are more favorably solvated than anions. For the smaller solutes, *ψ* increases with *q*, a signature of non-linear response. Anions consequently become more favorably solvated at large *q*. For the larger solutes (*R* = 0.75 nm and *R* = 1.0 nm) the solvent response is approximately linear, as reflected by the weak dependence of *ψ* on *q*.

Previous work has demonstrated that water's response to charging sub-nanometer cavities is significantly nonlinear.^24,31–34,36,37,50,55,56^ In *ψ*(*q*, *R*) the breakdown of linear dielectric behavior is evidenced by deviations away from the limiting value *ψ*(0, *R*) = *ϕ*_neut_(*R*). Fig. 2a shows our numerical results for the asymmetry potential as a function of *q* for solutes in bulk liquid SPC/E water. For large solutes (*R* ≳ 0.5 nm), the variation of *ψ* is modest as *q* increases from 0 to *e*. For smaller cavities, linear response theory fails dramatically, in that charge asymmetry changes many-fold as the solute is charged. In the case of a fluoride-sized solute, the asymmetry at full charge (*eψ*(*e*, *R*_F_) ≈ 26*k*_B_*T*) is qualitatively different than in linear response (*eϕ*_neut_(*R*_F_) ≈ −8*k*_B_*T*). For SPC models of bulk liquid water, the ultimate electrostatic bias in solvating cations and anions of this size clearly cannot be attributed to the innate environment of a neutral cavity, much less to the structure of a distant interface. *Ab initio* molecular dynamics studies have reached a similar conclusion.^31^

SPC simulations of bulk liquid water indicate that the nonlinearity of solvent response to solute charging has a step-like character:^33,34,36^ For one range of solute charge (*q* < *q*_c_), the susceptibility d〈*ϕ*_solv_〉_*q*_/d*q* is approximately constant. In the remaining range (*q* ≥ *q*_c_), d〈*ϕ*_solv_〉_*q*_/d*q* is also nearly constant, but with a different value. Piecewise linear response (PLR) models inspired by this observation give a broadly reasonable description of bulk solvation thermodynamics throughout the entire range −*e* < *q* < +*e*. In our discussion of ion adsorption below, we will assess the suitability of a PLR model for interfacial solvation as well.

## 2 Charge asymmetry in ion adsorption

In bulk liquid water, an electric potential from its bounding interfaces cannot be unambiguously identified. Even the sign of the bias generated by a liquid–vapor interface is unclear. Moreover, the nonlinear local response to solute charging can exert a bias on ion solvation that significantly outweighs the charge asymmetry due to distant interfaces.

Solvation within the interfacial environment is hardly less complex, juxtaposing the fluctuating intermolecular arrangements of bulk water together with broken symmetry and the microscopic shape variations of a soft boundary. It is thus unlikely that complications described in Section 1 for bulk liquid are much eased in the interfacial scenario. We should not expect, for example, that the neutral cavity potential for a solute positioned near the interface will be dominated by a simple nonlocal contribution. Nor should we expect the accuracy of linear response approximations to be greatly improved, such that *ϕ*_neut_ is predictive of charge asymmetric solvation.

The adsorption of an ion to the interface, however, concerns the *difference* in solvation properties of bulk and interfacial environments. To the extent that nonlinear response and local structuring at the interface are similar to those in bulk liquid, their effects may cancel, or at least significantly offset, in the thermodynamics of adsorption. Our main results concern this possibility of cancellation, which would justify regarding macroscopically nonlocal contributions to *ϕ*_neut_ as the basic origin of charge asymmetry in ion adsorption.

**Fig. 3:**
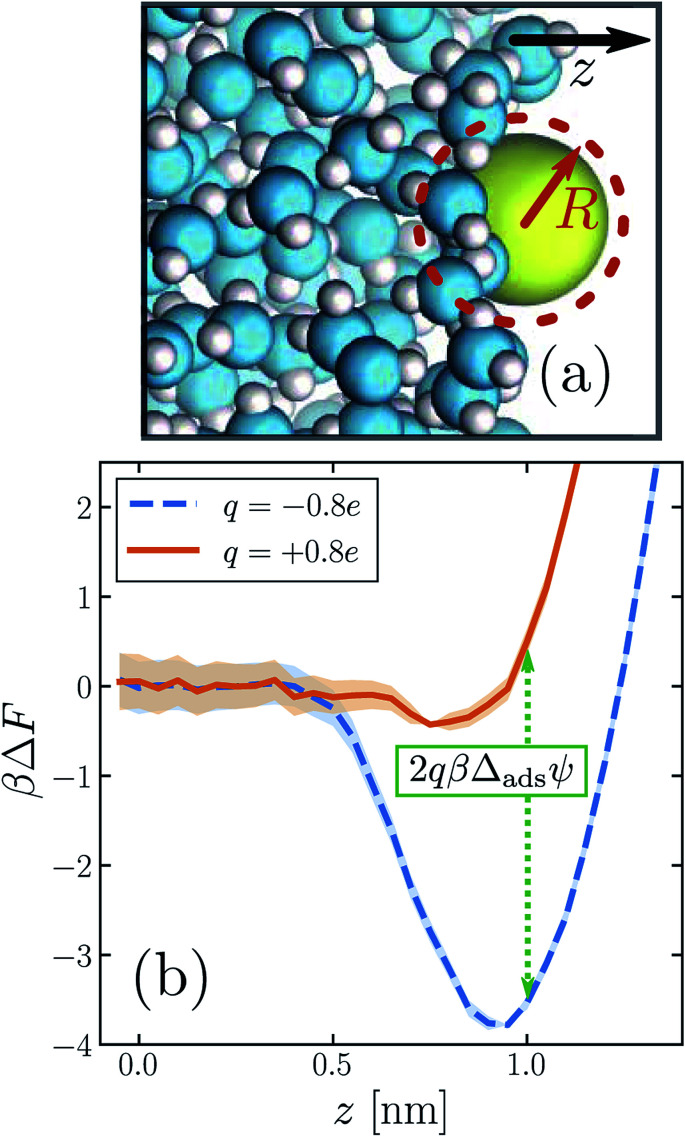
The propensity for an ion to adsorb to the air–water interface depends strongly on the sign of its charge. (a) Snapshot of an iodide-sized anion (*R* = 0.415 nm) at the interface. The system comprises a free-standing slab of liquid water surrounded on either side by its vapor. (Only one of the two interfaces is shown.) The *z* direction is indicated by the arrow. The size of the solute is depicted schematically by the dashed circle. (b) Potential of mean force Δ*F* as a function of ion position *z*, for a solute charge *q* = +0.8*e* (solid orange) and *q* = −0.8*e* (dashed blue). The anion adsorbs much more strongly to the interface than the cation for this solute size. The dotted green line indicates the connection between these free energy profiles and the adsorption asymmetry potential in eqn (6).

We begin by establishing that biases on solvation at the interface are complicated in ways that qualitatively resemble biases in bulk. As before, we consider solutes with a range of sizes and charges, now positioned at the liquid's boundary (illustrated in Fig. 3a). The free energies and potentials defined in Section 1 for bulk solution now acquire dependence on the Cartesian coordinate *z* that points perpendicular to the mean surface. ESI† shows the detailed location *z*_int_ we designate as adsorbed for each ion. In all cases *z*_int_ lies near the Gibbs dividing surface, where the solvent density falls to half its bulk value. The larger solutes occupy considerable volume, so that the solvent density profile in our finite simulation cell changes noticeably with their height *z*. A precise interfacial solute location is therefore difficult to justify. When neutral and located near *z*_int_, however, these nanometer-size solutes tend to deform the instantaneous phase boundary,^57–59^ just as they induce local drying in bulk solution.^49^ This response essentially fixes their location relative to the instantaneous interface, so that their solvation properties should be fairly insensitive to the choice of *z*_int_.

The neutral cavity potential for interfacial solutes is shown in Fig. 1. As was observed for the bulk liquid, *ϕ*_neut_ is consistently negative over the range *R* = 0.24 nm to *R* = 1 nm but varies significantly with solute size. In this case the potential increases nearly monotonically with *R*, though the values of *ϕ*_neut_(0.75 nm) and *ϕ*_neut_(1 nm) are statistically indistinguishable within our sampling. Just as for bulk liquid, we expect *ϕ*_neut_ to vanish in the limit *R* → ∞. Here, drying at the surface of very large solutes effects a distortion of the liquid–vapor interface that places the probe (located at the cavity's center) distinctly in the vapor phase. Judging from our results, the asymptotic approach to this limit is quite slow for interfacial solutes. Nonetheless, *ϕ*_neut_ changes by nearly 40% over the range of *R* considered, emphasizing the importance of local, solute-dependent contributions. As concluded for the bulk solvent, macroscopically nonlocal potentials arising from orientational structure of the air–water interface do not dominate the charge asymmetry experienced by neutral solutes at *z*_int_.

The response to charging a solute at the air–water interface is strongly nonlinear, to a degree comparable with bulk response. A similarly important role of nonlinear response at interfaces has been reported previously.^11,24^ The resulting *q*-dependent charge asymmetry closely resembles bulk behavior, as quantified by the asymmetry potential *ψ*(*q*, *R*; *z*), whose dependence on solute position we now make explicit. Fig. 2b shows simulation results for *ψ*(*q*, *R*; *z*_int_) for SPC/E water. On the scale that *ψ* changes as *q* increases from 0 to *e*, the charging response in bulk liquid and at the interface are nearly indistinguishable by eye. This close similarity suggests that the predominant source of nonlinearity lies in aspects of local response which are not so different in the two environments.

Comparing *ψ*(*q*, *R*; *z*_int_) with *ψ*(*q*, *R*; *z*_liq_), and *ϕ*_neut_(*R*; *z*_int_) with *ϕ*_neut_(*R*; *z*_liq_), gives a sense for features of solvation that most strongly shape ion adsorption. Similarities point to aspects of solvent structure and response which are largely unchanged when an ion moves to the interface. These contributions may be important for solvation in an absolute sense, but their cancellation indicates a weak net influence on adsorption thermodynamics.

For all values of *R* we considered, *ϕ*_neut_ is less negative at *z*_int_ than at *z*_liq_. In the simplest conception of the liquid's boundary as a layer of nonzero dipole density, one would expect the nonlocal component of *ϕ*_neut_ to attenuate steadily in magnitude as a solute moves from the liquid phase into the interfacial region, and then vanish as the solute enters vapor. Whether this rough picture is consistent with the observed shift in *ϕ*_neut_ depends on the sign of the nonlocal potential *ϕ*_d_. Unfortunately this sign is uncertain, as described in Section 1, due to the intrinsic ambiguity in dividing molecules between bulk and surface regions. Ref. 41 calculated a positive dipole component of the surface potential, *ϕ*_d_ = +260 mV. Within the simple continuum picture, this value suggests a downward shift in *ϕ*_neut_ as *z* increases from *z*_liq_ to *z*_int_, in contrast to our simulation results. A different partitioning scheme, however, can give *ϕ*_d_ < 0, suggesting an upward shift, as we observe in simulation.

Although the direction of change in *ϕ*_neut_ might be anticipated from the sign of *ϕ*_d_, the magnitude of this shift varies considerably with solute size. For *R* = 0.24 nm, |*ϕ*_neut_| is reduced by about 15% when the cavity is placed at the interface. For *R* = 0.415 nm the reduction is greater than 50%. This variation cannot arise from nonlocal biases, which are insensitive to the size or charge of a solute. A distinct, macroscopically nonlocal contribution could manifest as a nonzero asymptotic value of *Δ*_ads_*ϕ*_neut_ = *ϕ*_neut_(*R*; *z*_int_) − *ϕ*_neut_(*R*; *z*_liq_) at intermediate *R*; according to our data, if such a limit exists it occurs for solutes larger than 1 nm.

The similarity between the asymmetry potentials *ψ*(*q*, *R*) for solutes in the bulk and at the interface offers some hope that complicating factors of nonlinear response cancel out in the adsorption process. The extent of this cancellation is quantified by an adsorption asymmetry potential

**Fig. 4:**
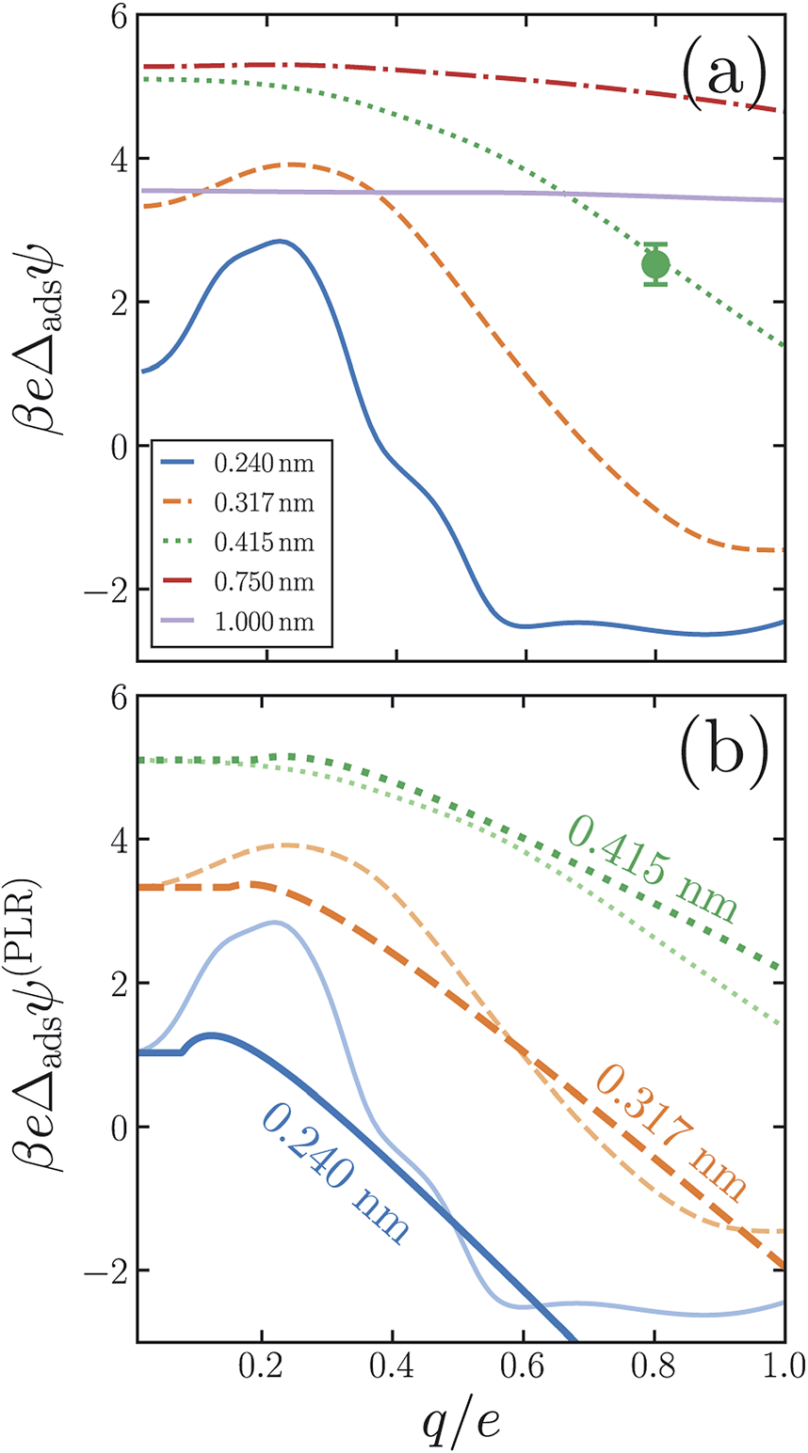
Linear response theory cannot faithfully describe the differences between adsorption profiles of sub-nanometer anions and cations, as demonstrated in (a) by variations in adsorption asymmetry potential *Δ*_ads_*ψ* with both *R* and *q*. For the smallest solutes (*R* ≲ 0.4 nm), *Δ*_ads_*ψ* even changes sign as *q* increases. In this size range, fully charged cations are more abundant at the interface than anions (with the same bulk concentration). At larger *R*, solutes with *q* = −*e* adsorb more strongly than those with *q* = +*e*. As the solute diameter approaches *R* = 1 nm, nonlinear response during the charging process becomes much less pronounced. Values of *R* are indicated in the legend. (b) A PLR model (heavy lines) predicts *Δ*_ads_*ψ* is initially flat, followed by a steady decrease as *q* increases. This qualitatively captures the simulation data (light lines), although it fails to capture the leveling off at large *q* seen for *R* = 0.240 nm and 0.317 nm.

5 *Δ*_ads_*ψ*(*q*,*R*) = *ψ*(*q*,*R*;*z*_int_) − *ψ*(*q*,*R*;*z*_liq_),

6
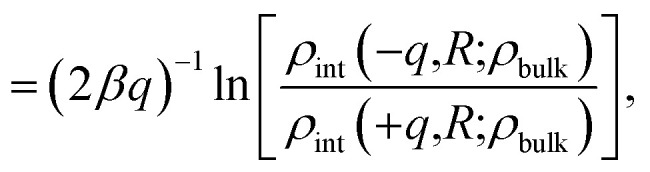
where *ρ*_int_ is the average number density of a solute at *z* = *z*_int_, given its concentration *ρ*_bulk_ in bulk solution. Eqn (6) highlights the direct relationship between *Δ*_ads_*ψ*(*q*, *R*) and the relative adsorption propensities of cations and anions: for dilute solutes with opposite charge, equal size, and equal bulk concentration, exp[2*βqΔ*_ads_*ψ*(*q*, *R*)] directly indicates the enhancement of anions over cations at the interface, as shown in Fig. 3 and 4. From the preceding discussion of the asymmetry potential itself, it is clear that *Δ*_ads_*ψ*(*q* → 0, *R*) = *Δ*_ads_*ϕ*_neut_. The full dependence of *Δ*_ads_*ψ* on *q* thus incorporates the adsorption behavior of the neutral cavity potential as well as the corresponding solvent response to charging. Our numerical results for *Δ*_ads_*ψ*(*q*, *R*) are the central contribution of this paper.

The adsorption asymmetry potential *Δ*_ads_*ψ*(*q*, *R*), as determined from simulations of the SPC/E model, are plotted as a function of *q* in Fig. 4 for several values of *R*. For the smaller solutes, the scale on which *Δ*_ads_*ψ* changes upon charging is dramatically smaller than the asymmetry potentials themselves. Nonlinear solvent response in these cases cancels substantially in the process of adsorption, but by no means completely. Despite the partial cancellation, *Δ*_ads_*ψ* still varies by more than 100 mV as *q* increases from 0 to *e*, comparable in magnitude to *ϕ*_d_ and *ϕ*_neut_. For *R* = 0.24 nm and *R* = 0.317 nm, this variation is sufficient to change even the *sign* of *Δ*_ads_*ψ*, and therefore to change the sense of charge bias: small monovalent cations “adsorb” more favorably to the air–water interface than do anions of the same size. In this size range, however, the adsorbed state is unstable relative to the fully solvated ion in bulk solvent unless *q* is very small in magnitude.

As was previously observed for bulk solvation, we find that the response to charging a solute at the air–water interface, while nonlinear on the whole, is roughly piecewise linear. Deviations from piecewise linearity are generally stronger in the interfacial case. It is therefore less straightforward to parameterize an interfacial piecewise linear response model, *i.e.*, to identify a crossover charge *q*_c_ at which the susceptibility d〈*ϕ*_solv_〉_*q*_/d*q* changes discontinuously. The ESI† presents plausible choices for *q*_c_ and these limiting susceptibilities for our three smallest solutes, from which adsorption asymmetry potentials *Δ*_ads_*ψ*^(PLR)^ can be readily computed. The resulting PLR predictions are plotted in Fig. 4b. Two basic features of our simulation results are accurately captured by this phenomenological description. Specifically, (i) for small solute charge, *Δ*_ads_*ψ* is an approximately constant or modestly increasing function of *q*, and (ii) a more strongly decreasing trend of *Δ*_ads_*ψ* follows for larger *q*. Nearly quantitative agreement can be obtained for an iodide-sized solute, *R* = 0.415 nm. Smaller solutes exhibit a more complicated charge dependence that lies beyond a simple PLR description. We note that this test of PLR is a demanding one, given the small scale of *Δ*_ads_*ψ* relative to *ψ*(*q*, *R*; *z*_int_) and *ψ*(*q*, *R*; *z*_liq_) individually. To the extent that PLR is a successful caricature, these results suggest that the adsorption charge asymmetry at full charging (*q* = *e*) derives from a combination of features of solvent response, including an interface-induced shift in the crossover charge *q*_c_ at which the character of linear response changes. The neutral cavity potential *ϕ*_neut_ figures into this combination as well, but by no means does it dominate for these solute sizes.

For the larger solutes we examined, the nonlinearity of solvent response to charging is not pronounced, either in bulk liquid or at the interface. The difference in nonlinearity of these environments is necessarily also not large, with *eΔ*_ads_*ψ* changing by less than *k*_B_*T* over the range *q* = 0 to *q* = *e*. This small variation is comparable in scale to those of *ψ*(*q*, *R*; *z*_int_) and *ψ*(*q*, *R*; *z*_liq_) themselves. Judged on that scale, the cancellation of nonlinear response is in fact less complete for *R* = 0.75 nm than for smaller solutes. As we have discussed, cavities with *R* ≳ 1 nm depress the instantaneous interface significantly, effectively placing them in the vapor phase even when *z* coincides with the Gibbs dividing surface. When such a solute is endowed with sufficient charge, wetting will occur at its surface, eventually raising the interface to effectively move the solute into the liquid phase. This solvent response, which originates in the physics of phase separation, is intrinsically nonlinear. For large *R*, a solute charge well in excess of *e* is required to fully induce this structural change, but at the nanoscale it may manifest as an incipient nonlinearity for *q* ≈ *e*.

In summary, the adsorption asymmetry potential *Δ*_ads_*ψ* depends significantly on solute size *R* and charge *q*. Neither of these sensitivities can arise from intrinsic orientational bias at the neat air–water interface. Long-range electrostatic forces from oriented molecules at the liquid's boundary, which contribute importantly to surface potentials like *ϕ*_surf_ and *ϕ*_d_, are inherently unaffected by the presence, size, or charge of a sufficiently distant solute. These results highlight the importance of local solvent structure and response for charge asymmetry in interfacial ion adsorption, and they highlight the danger of inferring solvation thermodynamics from ion-free quantities such as *ϕ*_surf_ and *ϕ*_d_.

## 3 Discussion and conclusions

In this study, we set out to understand whether or not charge asymmetry in interfacial ion adsorption could be understood in terms of macroscopically long-ranged, collective forces intrinsic to water. For ion solvation in bulk, difficulties in unambiguously determining such long-ranged contributions were already apparent from previous results. Building on that work, our results show that for SPC models of water such a simple mechanistic picture is inadequate for interfacial solvation as well. In addition to the difficulties in partitioning molecules between ‘near’ and ‘distant’ interfaces, complex nonlinear response also underlies substantial shortcomings of trying to rationalize ion adsorption from surface potentials that characterize biases of the undisturbed air–water interface. The nonlinearities in *F*_chg_(*q*, *R*) for bulk and interfacial environments, while similar, are sufficiently different that the process of adsorption is also substantially nonlinear. A compelling inference of adsorption tendencies from intrinsic properties of the undisturbed liquid and its interface with vapor requires information that is more subtle than an average electric potential and macroscopic dielectric susceptibility. As highlighted by the potential distribution theorem,^60^ this information can in principle be gleaned from equilibrium statistics of the undisturbed solvent. But in terms of fluctuations in electric potential, it involves high-order correlations whose physical meaning is not transparent.

In previous work we developed and tested finite size corrections for computer simulations of interfacial ion solvation.^37^ Based on DCT, these corrections proved to be quite accurate even for simulation unit cells with nanometer dimensions. Our conclusion that DCT is a faithful representation of aqueous polarization response down to nanometer length scales is reinforced by the results of this paper. In particular, when charging a solute of diameter *R* = 1 nm, solvent response on an absolute scale is linear to a very good approximation, both in bulk liquid and at the interface. The results of Fig. 2 and 4, however, also indicate that 1 nm marks the validity limit of linear response. When charging a cavity with *R* = 0.75 nm, nonlinear contributions to charge asymmetry are quantitatively important; for smaller solutes such nonlinear contributions become not just important but instead dominant. In passing, we note that even for the larger solutes, a significant charge asymmetry persists, both for bulk solvation and for adsorption to the interface. This persistent bias weighs against the basis of the tetra-phenyl arsonium/tetra-phenyl borate (‘TATB’) extrathermodynamic assumption, an issue that has also been raised by others.^39,61–63^

The highly simplified description of molecular interactions in SPC models is certainly a crude approximation to real microscopic forces. But the specific ion effects it exhibits cannot be ascribed simply to an errant surface potential. Indeed, discrepancies between models in potentials such as *ϕ*_d_ (whose definition requires an arbitrary convention), *ϕ*_surf_ (which pertains to a solute that does not exclude volume), or even *ϕ*_neut_ (which for subnanometer solutes does not account for the strong asymmetry of solvent response) are not greatly alarming. *ϕ*_surf_ and *ϕ*_d_ can vary significantly among different models, but they do not weigh on ion solvation thermodynamics in a direct way, either in bulk liquid or at the air–water interface. (This does not contradict their use for computing *F*_chg_ once a choice for partitioning molecules between the interface and bulk has been made.) By contrast, trends in *F*_solv_ and *Δ*_ads_*ψ* at full charging reflect on essential microscopic mechanisms that underlie specific ion adsorption. SPC models may be best viewed as caricatures of a disordered tetrahedral network, with intrinsic charge asymmetry due to the distinct geometric requirements of donating and accepting hydrogen bonds. These essential features of liquid water are often associated with nonlinear response in solvation.^64–66^ By implicating nonlinearities of precisely this kind as sources of ion-specific adsorption properties, our results support the use of SPC models as a physically motivated test bed for exploring the microscopic basis of surprising trends in interfacial solvation. Conversely, our results underscore the limitations of DCT and notions of long-ranged contributions from unperturbed interfaces, which do not describe essential local aspects of the chemical physics underlying ion adsorption and its charge asymmetry. The consequences of this shortcoming are likely to be exacerbated in confined geometries. Work to move beyond standard DCT approaches is an active area of research (*e.g.*ref. 24–26 and 67) and it is hoped that the results presented in this study will help to guide future theoretical developments.

## 4 Methods

All simulations used the SPC/E water model;^40^ solutes were represented as Lennard-Jones (LJ) spheres with a central charge *q*. The SHAKE algorithm was used to maintain a rigid water geometry.^68^ Periodic boundary conditions were imposed in all three Cartesian directions, with the liquid phase spanning two directions in a slab geometry. Long-range Coulomb interactions were summed using the particle–particle particle-mesh Ewald method.^69,70^ A spatially homogeneous background charge was included to maintain electroneutrality and thus guarantee finite electrostatic energy. For solute sizes *R* < 0.75 nm, the system comprised 266 water molecules with simulation cell dimensions 2 × 2 × 4.5 nm^3^. For *R* ≥ 0.75 nm the simulation cell size was 3.5 × 3.5 × 8.5 nm^3^ and we used 1429 water molecules. Solvent density profiles that indicate the interfacial location *z*_int_ for each solute are given in the ESI.† A time step of 1 fs was used for all simulations. A temperature of 298 K was maintained using Langevin dynamics,^71,72^ as implemented in the LAMMPS simulation package,^73^ which was used throughout.

Due to the long range of Coulomb interactions, ion solvation in polar solvents has important contributions even from distant solvent molecules. Thermodynamic estimates from molecular simulations are thus subject to substantial finite size effects, which have been the focus of many studies.^34,74–77^ In ref. 37 we showed for liquid water in a periodic slab geometry that values of *ϕ*_neut_ depend on simulation box size in a slowly decaying but predictable way. The limit of infinitely separated periodic images can thus be obtained with a simple finite size correction, which amounts to referencing electric potential values to the vapor phase. We have applied this correction to all potentials reported in this paper. The potential of mean force Δ*F*(*q*, *R*; *z*) for ions in periodic liquid slabs are, by contrast, nearly independent of simulation cell size for *z* ≤ *z*_int_.^37^

To compute Δ*F*(*q*, *R*; *z*), we followed the same procedure as outlined in ref. 18, namely umbrella sampling and histogram reweighting with MBAR.^78^ To calculate *ψ*(*q*, *R*; *z*) for a given choice of *R* and *z*, simulations were performed with *q*/*e* = −1.0, −0.9, …, +0.9, and +1.0. This spacing of *q* values allows for ample overlap among probability distributions *P*_*q*_(*ϕ*_solv_) of the electrostatic potential at the center of the solute (Fig. S10†). For *R* ≤ 0.415 nm statistics were obtained from trajectories 5 ns in duration. For *R* = 0.75 nm and 1.0 nm, trajectories varied between 2.8 ns and 5.0 ns. Using the MBAR algorithm, results from the entire range of solute charge were combined to determine the neutral cavity distribution *P*_0_(*ϕ*_solv_) over a correspondingly broad range of *ϕ*_solv_. *F*_chg_ was computed by averaging exp(−*βqϕ*_solv_) according to the distribution *P*_0_(*ϕ*_solv_), as prescribed by Widom's potential distribution theorem,^60^7
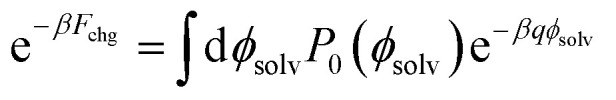


The integral in eqn (7) was performed numerically.

## Conflicts of interest

There are no conflicts to declare.

## Supplementary Material

SC-011-D0SC01947J-s001
