# Supplementary material for: Assessing long-range contributions to the charge asymmetry of ion adsorption at the air–water interface
Source: Chem Sci. 2020 Oct 5;11(43):11791–800. doi: 10.1039/d0sc01947j (PMC8162909; doi:10.1039/d0sc01947j)
Supplement: SC-011-D0SC01947J-s001 [file SC-011-D0SC01947J-s001.pdf]

Electronic Supplementary Material (ESI) for Chemical Science.
This journal is © The Royal Society of Chemistry 2020

Supporting information for "Assessing long-range contributions to the charge asymmetry of ion adsorption at the air-water interface"

Stephen J. Cox,[1] Dayton G. Thorpe,[2,3] Patrick R. Shaffer,[4] and Phillip L. Geissler[2,4]

[1] *Department of Chemistry, University of Cambridge, Lensfield Road, Cambridge CB2 1EW, United Kingdom*

[2] *Chemical Sciences Division, Lawrence Berkeley National Laboratory, Berkeley, CA 94720, United States.*

[3] *Department of Physics, University of California, Berkeley, CA 94720, United States.*

[4] *Department of Chemistry, University of California, Berkeley, CA 94720, United States.*

(Dated: 1 October 2020)

This document contains a detailed account of the issues faced when trying to isolate contributions to $\phi_{\rm neut}$ from local and distant sources. Also given are solvent density profiles $\rho(z)$ in the presence of the neutral solute for the different systems studied, and the position of the solute at the interface is indicated in each instance. Solute-solvent radial distribution functions $g(r)$ are shown for $q = -e$, 0 and $+e$ with the solute in the center of the slab. Details underlying the piecewise linear response model are also presented. A brief description of how $P_0(\phi_{\rm solv})$ is obtained is given.

I. ELECTROSTATIC CONTRIBUTIONS FROM NEAR AND FAR

The challenge of identifying and interpreting a potential drop across the liquid-vapor interface can be viewed as an issue of partitioning molecules between distinct regions of space.

Consider a macroscopic droplet of liquid bounded by an interface S with the vapor phase (as illustrated in Fig. S1). The origin of our coordinate system lies deep within the bulk liquid phase. We will aim to calculate the average electric potential $\langle\phi\rangle$ at the origin, distinguishing contributions of molecules that are far from the probe (including those at the phase boundary) from those that lie nearer the origin. Specifically, we will divide the two populations at an imaginary surface B that is also deep within the bulk liquid. We will take B to be distant enough from the origin that liquid structure on this surface is bulk in character, even if the microscopic vicinity of the origin is complicated by a solute's excluded volume.

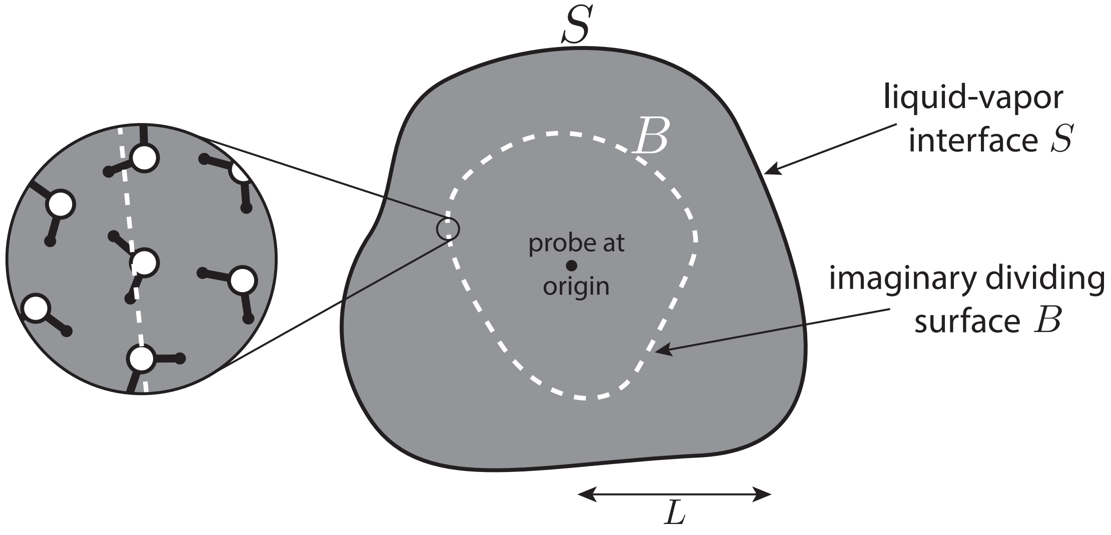

FIG. S1. Sketch of a macroscopic droplet of liquid (shaded region at right) surrounded by dilute vapor. The two phases contact at a macroscopically smooth interface S. The surface B within the droplet is a mathematical device to isolate the electrostatic contribution of molecules residing near the phase boundary S. The droplet's overall scale L is a macroscopic distance. A magnified view of a microscopic region straddling B is shown at left. Molecules intersected by B (dashed white line) could reasonably be assigned to either the near (inside B) or far (outside B) domains.

A. Partitioning schemes

The vast majority of molecules in the droplet are unambiguously located either outside B ("far") or inside B ("near"). A tiny fraction straddle the surface B. In the case of water this could involve a molecule's oxygen atom lying on one side of B, while its hydrogen atoms lie on the other. One division scheme (an M-scheme) would judge the molecule's location based on the O atom; another M-scheme might base the classification on the molecule's center of charge. A still different scheme (a P-scheme) could divide the molecule in two, with some pieces "near" and other pieces "far". (The M-scheme and the P-scheme are well known in the literature. See e.g. Ref. 1.) The total potential ϕ at the probe site is not sensitive to which of these schemes is chosen. But its contributions ϕ_{near} and ϕ_{far} from atoms/molecules in the near and far regions *are* sensitive, in an offsetting way.

Let's first treat the M scheme, with the molecule's near/far classification based on the position $\mathbf{r}^{(0)}$ of some site within the molecule (say, its O atom). The average far-field potential in this case is

$$\langle \phi_{\text{far}}^{\text{M}} \rangle = N \int_{\text{outside } B} d\mathbf{r} \int d\boldsymbol{\Omega}\, p(\mathbf{r}, \boldsymbol{\Omega}) \sum_{\alpha} \frac{q_\alpha}{|\mathbf{r} + \Delta \mathbf{r}_\alpha(\boldsymbol{\Omega})|}, \tag{S1}$$

where N is the total number of molecules in the droplet and α indexes charged sites within each molecule. Here, $p(\mathbf{r}, \boldsymbol{\Omega}) = \langle \delta(\mathbf{r} - \mathbf{r}^{(0)}) \delta(\boldsymbol{\Omega} - \boldsymbol{\Omega}^{(0)}) \rangle$ is the joint probability distribution of a molecule's position (i.e., $\mathbf{r}^{(0)}$) and intramolecular configuration $\boldsymbol{\Omega}^{(0)}$ (specified relative to the reference position $\mathbf{r}^{(0)}$, as indicated by the superscript).[2] By $\Delta \mathbf{r}_\alpha = \mathbf{r}_\alpha - \mathbf{r}^{(0)}$ we denote the displacement of charge q_α from the reference point $\mathbf{r}^{(0)}$. This intramolecular displacement is entirely determined by $\boldsymbol{\Omega}^{(0)}$.

For the P-scheme, each charge α contributes to $\phi_{\text{far}}^{\text{P}}$ if $\mathbf{r}_\alpha$ lies outside B. The corresponding far-field potential is

$$\langle \phi_{\text{far}}^{\text{P}} \rangle = N \sum_{\alpha} \int_{\text{outside } B} d\mathbf{r} \int d\boldsymbol{\Omega}\, p_\alpha(\mathbf{r}, \boldsymbol{\Omega}) \frac{q_\alpha}{|\mathbf{r}|} \tag{S2}$$

$$= N \sum_{\alpha} \int_{\text{outside } B} d\mathbf{r} \int d\boldsymbol{\Omega}\, p(\mathbf{r} - \Delta \mathbf{r}_\alpha, \boldsymbol{\Omega}) \frac{q_\alpha}{|\mathbf{r}|} \tag{S3}$$

where $p_\alpha(\mathbf{r}, \boldsymbol{\Omega})$ is the joint probability distribution for site position $\mathbf{r}_\alpha$ and intramolecular configuration of a solvent molecule. In Eq. S3 we have made use of the connection

$$p_\alpha(\mathbf{r}, \boldsymbol{\Omega}) = \langle \delta(\mathbf{r} - \mathbf{r}_\alpha) \delta(\boldsymbol{\Omega} - \boldsymbol{\Omega}^{(0)}) \rangle = \langle \delta(\mathbf{r} - \Delta \mathbf{r}_\alpha - \mathbf{r}^{(0)}) \delta(\boldsymbol{\Omega} - \boldsymbol{\Omega}^{(0)}) \rangle \tag{S4}$$

$$= p(\mathbf{r} - \Delta \mathbf{r}_\alpha(\boldsymbol{\Omega}), \boldsymbol{\Omega}) \tag{S5}$$

between the distributions p and p_α.

B. Multipole expansion

Since the entire "far" region is macroscopically distant from the origin, small-$\Delta\mathbf{r}_\alpha$ expansions of $|\mathbf{r}+\Delta\mathbf{r}_\alpha|^{-1}$ and $p(\mathbf{r}-\Delta\mathbf{r}_\alpha,\mathbf{\Omega})$ are well justified. These yield

$$\sum_\alpha \frac{q_\alpha}{|\mathbf{r}+\Delta\mathbf{r}_\alpha|} = \left(\sum_\alpha q_\alpha \Delta\mathbf{r}_\alpha\right)\cdot\nabla\frac{1}{r} + \frac{1}{2}\left(\sum_\alpha q_\alpha \Delta\mathbf{r}_\alpha\Delta\mathbf{r}_\alpha\right) : \nabla\nabla\frac{1}{r} + \ldots \tag{S6}$$

and

$$\sum_\alpha q_\alpha p(\mathbf{r}-\Delta\mathbf{r}_\alpha,\mathbf{\Omega}) = -\nabla\cdot\sum_\alpha q_\alpha\Delta\mathbf{r}_\alpha p(\mathbf{r},\mathbf{\Omega}) + \frac{1}{2}\nabla\nabla : \sum_\alpha q_\alpha \Delta\mathbf{r}_\alpha\Delta\mathbf{r}_\alpha p(\mathbf{r},\mathbf{\Omega}) + \ldots \tag{S7}$$

where we have omitted leading terms proportional to $\sum_\alpha q_\alpha$, which vanish by molecular charge neutrality. When carried through subsequent calculations, terms beyond quadrupole order in these expansions would vanish due either to symmetry or to the macroscopic scale of the droplet.

Defining dipole and quadrupole densities as

$$\mathbf{m}(\mathbf{r}) = N\int d\mathbf{\Omega}\, p(\mathbf{r},\mathbf{\Omega})\sum_\alpha q_\alpha\Delta\mathbf{r}_\alpha \tag{S8}$$

and

$$\mathbf{Q}(\mathbf{r}) = \frac{N}{2}\int d\mathbf{\Omega}\, p(\mathbf{r},\mathbf{\Omega})\sum_\alpha q_\alpha\Delta\mathbf{r}_\alpha\Delta\mathbf{r}_\alpha \tag{S9}$$

we can write

$$\langle\phi^{\mathrm{M}}_{\mathrm{far}}\rangle = \int_{\text{outside }B} d\mathbf{r}\left(\mathbf{m}(\mathbf{r})\cdot\nabla\frac{1}{r} + \mathbf{Q}(\mathbf{r}) : \nabla\nabla\frac{1}{r} + \ldots\right) \tag{S10}$$

and

$$\langle\phi^{\mathrm{P}}_{\mathrm{far}}\rangle = \int_{\text{outside }B} d\mathbf{r}\frac{1}{r}\left(-\nabla\cdot\mathbf{m}(\mathbf{r}) + \nabla\nabla : \mathbf{Q}(\mathbf{r}) + \ldots\right) \tag{S11}$$

Integrating by parts, and noting that $\mathbf{m}(\mathbf{r})$ and $\nabla : \mathbf{Q}(\mathbf{r})$ vanish both on B and at infinity,

$$\langle\phi^{\mathrm{P}}_{\mathrm{far}}\rangle = \int_{\text{outside }B} d\mathbf{r}\left(\mathbf{m}(\mathbf{r})\cdot\nabla\frac{1}{r} - \left(\nabla\frac{1}{r}\right)\cdot\left(\nabla\cdot\mathbf{Q}(\mathbf{r})\right)\right) \tag{S12}$$

$$= \int_{\text{outside }B} d\mathbf{r}\left(\mathbf{m}(\mathbf{r})\cdot\nabla\frac{1}{r} - \nabla\cdot\left(\nabla\frac{1}{r}\cdot\mathbf{Q}(\mathbf{r})\right) + \mathbf{Q}(\mathbf{r}) : \nabla\nabla\frac{1}{r}\right) \tag{S13}$$

Using the divergence theorem,

$$\langle \phi_{\text{far}}^{\text{P}} \rangle = \langle \phi_{\text{far}}^{\text{M}} \rangle - \int_B d\mathbf{R}\, \hat{\mathbf{n}}(\mathbf{R}) \cdot \left(\nabla \frac{1}{R} \cdot \mathbf{Q}(\mathbf{R}) \right) \tag{S14}$$

where $\mathbf{R}$ is a point on B and $\hat{\mathbf{n}}(\mathbf{R})$ is the corresponding local *inward*-pointing normal vector. Since B lies within the bulk liquid, where the average quadrupole density $\mathbf{Q}_{\text{liq}}$ is isotropic, $\mathbf{Q}(\mathbf{r}) = \mathbf{I}\,(\text{Tr}\mathbf{Q}_{\text{liq}}/3)$ everywhere on this surface. As a result,

$$\langle \phi_{\text{far}}^{\text{P}} \rangle = \langle \phi_{\text{far}}^{\text{M}} \rangle + \frac{\text{Tr}\mathbf{Q}_{\text{liq}}}{3} \int_{\text{inside } B} d\mathbf{r} \nabla^2 \frac{1}{r} \tag{S15}$$

$$= \langle \phi_{\text{far}}^{\text{M}} \rangle - \frac{4\pi}{3} \text{Tr}\mathbf{Q}_{\text{liq}} \tag{S16}$$

These two measures of the far-field potential are thus different. Moreover, the quadrupole trace that determines this difference depends on the choice of $\mathbf{r}^{(0)}$. This ambiguity is a well-known feature of the so-called Bethe potential $-(4\pi/3)\text{Tr}\mathbf{Q}_{\text{liq}}$.[3–8]

C. Dipole surface potential

To simplify the result for $\langle \phi_{\text{far}}^{\text{M}} \rangle$, note that $\mathbf{Q}(\mathbf{r})$ is isotropic everywhere outside B, except in the microscopic vicinity of S. In the bulk regions of the far domain, we then have $\mathbf{Q}(\mathbf{r}) : \nabla\nabla r^{-1} \propto \delta(\mathbf{r}) = 0$. The final term in Eq. S10 therefore has nonzero contributions only from a thin shell whose volume is proportional to L^2, where L is the macroscopic scale of the droplet. Since $\nabla\nabla r^{-1} \sim L^{-3}$ in this shell, the quadrupolar contribution to $\langle \phi_{\text{far}}^{\text{M}} \rangle$ has a negligible magnitude, L^{-1}. As a result,

$$\langle \phi_{\text{far}}^{\text{M}} \rangle = \int_{\text{outside } B} d\mathbf{r}\, \mathbf{m}(\mathbf{r}) \cdot \nabla \frac{1}{r} \tag{S17}$$

This integral similarly has nonzero contributions only from a microscopically thin shell of broken symmetry, centered on the phase boundary S. Since the macroscopic surface is very smooth on this scale, and because the average dipole density points normal to the locally planar interface, the far-field potential may be written

$$\langle \phi_{\text{far}}^{\text{M}} \rangle = \int_S d\mathbf{R} \int dz\, m_\perp(z)\, \hat{\mathbf{n}}(\mathbf{R}) \cdot \nabla \frac{1}{r}, \tag{S18}$$

where $\mathbf{R}$ is the point on S nearest to $\mathbf{r}$, the coordinate $z = (\mathbf{r} - \mathbf{R}) \cdot \hat{\mathbf{n}}(\mathbf{R})$ is the perpendicular displacement from the liquid-vapor interface, $\hat{\mathbf{n}}(\mathbf{R})$ is the outward-pointing normal of S, and

$m_\perp(z)\hat{\mathbf{n}}(\mathbf{R})$ is the average dipole field at $\mathbf{r}$. Neglecting contributions of $\mathcal{O}(z/L)$, we may replace r^{-1} by R^{-1}, and easily evaluate the surface integral, yielding

$$\langle \phi_{\text{far}}^{\text{M}} \rangle = -4\pi \int_{z_{\text{liq}}}^{z_{\text{vap}}} dz\, m_\perp(z), \tag{S19}$$

where the integral is performed in the direction from liquid ($z_{\text{liq}} < 0$) to vapor ($z_{\text{vap}} > 0$). For the case of a perfectly planar interface, this result is a familiar component of the surface potential, identified by Remsing et al. as the surface dipole contribution.[4,7] As they note, its value depends on the reference position $\mathbf{r}^{(0)}$ defining the molecular reference frame. In our calculation this dependence arises from the way molecules are classified relative to the dividing surface B.

D. Near-field potential

In evaluating $\langle \phi_{\text{far}} \rangle$, we have made no assumptions about the liquid's structure near the probe. If the origin lies inside a solute's excluded volume, then the near-field potential is complicated by the microscopically heterogeneous arrangement of solvent molecules in its vicinity. If, however, the probe is simply a point within the isotropic bulk liquid, then $\langle \phi_{\text{near}} \rangle$ can be easily determined.

For a probe that resides in uniform bulk liquid, $\mathbf{m}(\mathbf{r}) = 0$ and $\mathbf{Q}(\mathbf{r}) = \mathbf{Q}_{\text{liq}}$ everywhere inside B. In the P-scheme we can conclude immediately from the analogue of Eq. S11 that $\langle \phi_{\text{near}} \rangle = 0$. In the M-scheme we have

$$\langle \phi_{\text{near}}^{\text{M}} \rangle = \int_{\text{inside } B} d\mathbf{r}\, \frac{\text{Tr}\mathbf{Q}_{\text{liq}}}{3}\, \nabla^2 \frac{1}{r} = -\frac{4\pi}{3}\text{Tr}\mathbf{Q}_{\text{liq}} \tag{S20}$$

In either case the total potential sums to

$$\begin{aligned}
\langle \phi \rangle &= \langle \phi_{\text{near}}^{\text{M}} \rangle + \langle \phi_{\text{far}}^{\text{M}} \rangle && \text{(S21)}\\
&= \langle \phi_{\text{near}}^{\text{P}} \rangle + \langle \phi_{\text{far}}^{\text{P}} \rangle && \text{(S22)}\\
&= -4\pi \int_{z_{\text{liq}}}^{z_{\text{vap}}} dz\, m_\perp(z) - \frac{4\pi}{3}\text{Tr}\mathbf{Q}_{\text{liq}} && \text{(S23)}
\end{aligned}$$

These calculations of local and nonlocal contributions to the mean electrostatic potential resemble previous developments of surface potential in many ways.[4,5,7–12] Ours are somewhat more general than standard calculations, in that we do not require a specific shape of the liquid domain. (The standard development presumes an idealized geometry of the liquid

phase e.g. planar interface or spherical droplet, and integrates the resulting 1-dimensional Poisson equation.) More interestingly, it places the ambiguities surrounding surface potential in an easily conceived context: The electrostatic bias of an interface is not well defined because there is no unique way to assign molecules to that interface. Any attempt to do so carries an arbitrariness that (in the case of water) is comparable in magnitude to the apparent surface potential itself.

II. SOLVENT DENSITY PROFILES AND SOLUTE-SOLVENT RADIAL DISTRIBUTION FUNCTIONS

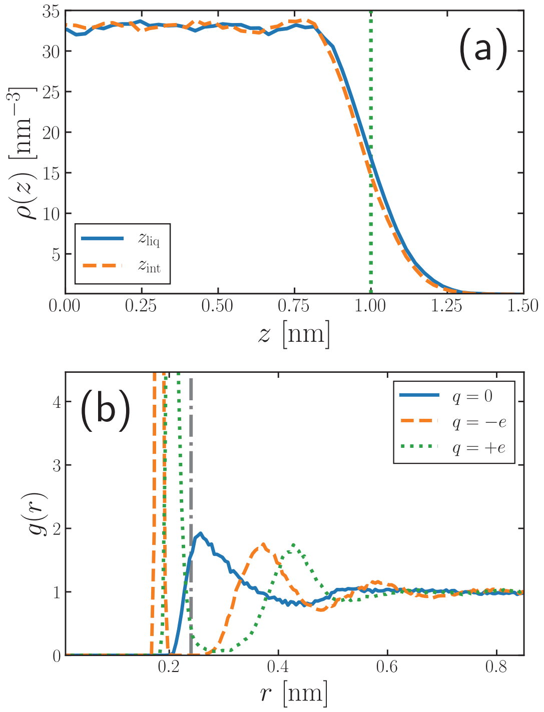

FIG. S2. (a) Average solvent density $\rho(z)$, plotted as a function of the coordinate z perpendicular to the liquid-vapor interface, with the solute ($R = 0.240\,\text{nm}$) located in the bulk ($z_{\text{liq}} = 0\,\text{nm}$, solid blue line), and at the interface ($z_{\text{int}} = 1\,\text{nm}$, dashed orange line). The dotted green line is drawn at $z = z_{\text{int}}$. Only half ($z > 0\,\text{nm}$) of the solvent profile is shown. (b) Radial distribution function $g(r)$, plotted as a function of the distance r between the solute's center and the oxygen atom of a water molecule, with the solute at $z = z_{\text{liq}}$ with $q = -e$, 0, and $+e$. The vertical dot-dashed gray line is drawn at $r = R$.

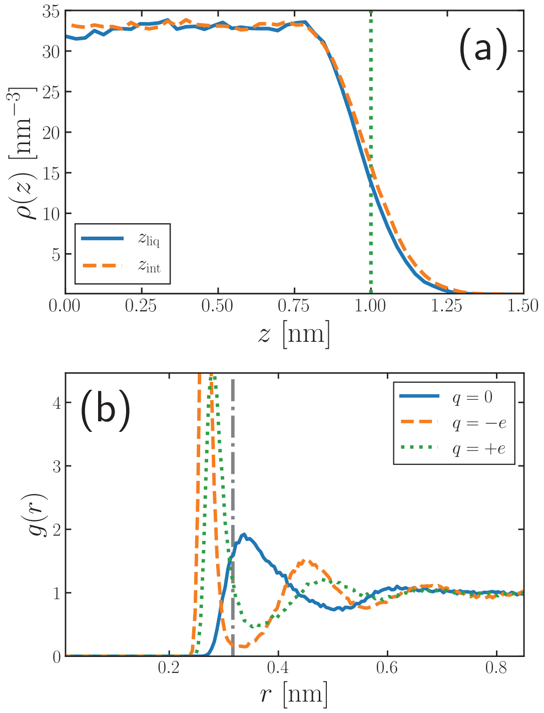

FIG. S3. (a) $\rho(z)$ with the solute ($R = 0.317\,\mathrm{nm}$) located in the bulk ($z_{\mathrm{liq}} = 0\,\mathrm{nm}$, solid blue line), and at the interface ($z_{\mathrm{int}} = 1\,\mathrm{nm}$, dashed orange line). The dotted green line is drawn at $z = z_{\mathrm{int}}$. Only half ($z > 0\,\mathrm{nm}$) of the profile is shown. (b) $g(r)$ with the solute at $z = z_{\mathrm{liq}}$ with $q = -e$, 0, and $+e$. The vertical dot-dashed gray line is drawn at $r = R$.

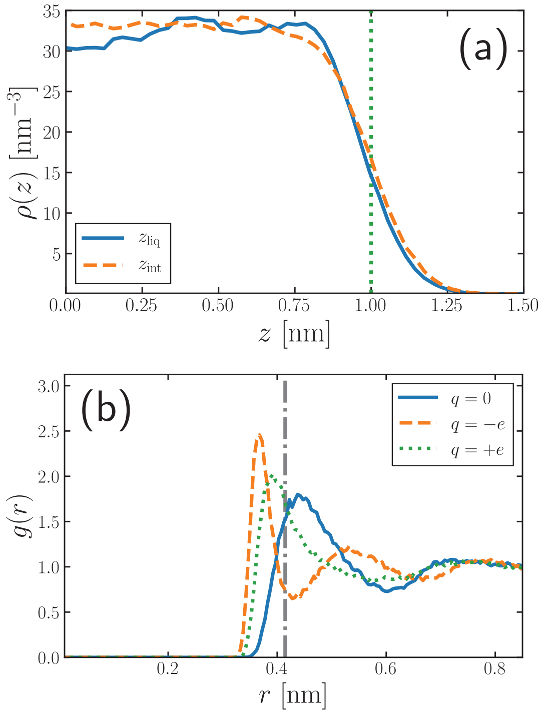

FIG. S4. (a) $\rho(z)$ with the solute ($R = 0.415\,\text{nm}$) located in the bulk ($z_{\text{liq}} = 0\,\text{nm}$, solid blue line), and at the interface ($z_{\text{int}} = 1\,\text{nm}$, dashed orange line). The dotted green line is drawn at $z = z_{\text{int}}$. Only half ($z > 0\,\text{nm}$) of the profile is shown. (b) $g(r)$ with the solute at $z = z_{\text{liq}}$ with $q = -e$, 0, and $+e$. The vertical dot-dashed gray line is drawn at $r = R$.

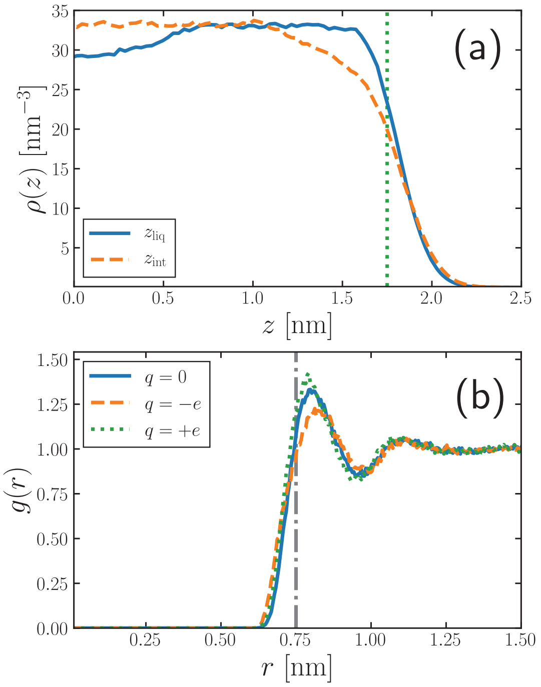

FIG. S5. (a) $\rho(z)$ with the solute ($R = 0.75\,\mathrm{nm}$) located in the bulk ($z_{\rm liq} = 0\,\mathrm{nm}$, solid blue line), and at the interface ($z_{\rm int} = 1.75\,\mathrm{nm}$, dashed orange line). The dotted green line is drawn at $z = z_{\rm int}$. Only half ($z > 0\,\mathrm{nm}$) of the profile is shown. (b) $g(r)$ with the solute at $z = z_{\rm liq}$ with $q = -e$, 0, and $+e$. The vertical dot-dashed gray line is drawn at $r = R$.

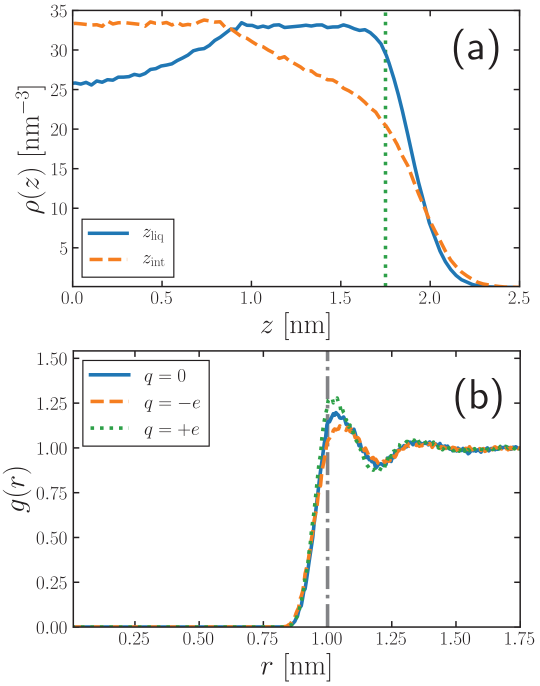

FIG. S6. (a) $\rho(z)$ with the solute ($R = 1.0\,$nm) located in the bulk ($z_{\rm liq} = 0\,$nm, solid blue line), and at the interface ($z_{\rm int} = 1.75\,$nm, dashed orange line). The dotted green line is drawn at $z = z_{\rm int}$. Only half ($z > 0\,$nm) of the profile is shown. (b) $g(r)$ with the solute at $z = z_{\rm liq}$ with $q = -e$, 0, and $+e$. The vertical dot-dashed gray line is drawn at $r = R$.

III. EVALUATING PIECEWISE LINEAR RESPONSE

A. Outline

Here we present details of the piecewise linear response (PLR) model discussed in the main article. The PLR model is based on the observation that solvent response to charging a solute is linear for both anions and cations, but differs between the two cases.[13–15] In such a model, the average electrostatic potential due to the solvent at the center of a charged cavity can be written as

$$\langle\phi_{\rm solv}\rangle_q = \begin{cases} \phi_{\rm neut} - \beta q\langle(\delta\phi_{\rm solv})^2\rangle_+ & (q \geq q_{\rm c}) \\ \phi_{\rm neut} - \beta q\langle(\delta\phi_{\rm solv})^2\rangle_- - \beta q_{\rm c}\left[\langle(\delta\phi_{\rm solv})^2\rangle_+ - \langle(\delta\phi_{\rm solv})^2\rangle_-\right] & (q < q_{\rm c}), \end{cases} \tag{S24}$$

where $q_{\rm c}$ is the value of the 'crossover charge' between the two linear regimes, $\langle(\delta\phi_{\rm solv})^2\rangle_+$ is the variance of $\phi_{\rm solv}$ for $q \geq q_{\rm c}$, and $\langle(\delta\phi_{\rm solv})^2\rangle_-$ is the variance of $\phi_{\rm solv}$ for $q < q_{\rm c}$. (As written, it is implicitly assumed that $q_{\rm c} \leq 0$, as suggested by simulations.) Let us define $J = [\langle(\delta\phi_{\rm solv})^2\rangle_+ - \langle(\delta\phi_{\rm solv})^2\rangle_-]$. $F_{\rm chg}$ is then,

$$F_{\rm chg}(q) = \begin{cases} q\phi_{\rm neut} - \frac{\beta q^2}{2}\langle(\delta\phi_{\rm solv})^2\rangle_+ & (q \geq q_{\rm c}) \\ q\phi_{\rm neut} - \frac{\beta q^2}{2}\langle(\delta\phi_{\rm solv})^2\rangle_- - \beta J\left(qq_c - \frac{q_c^2}{2}\right) & (q < q_{\rm c}), \end{cases} \tag{S25}$$

and ψ is,

$$\psi(q) = \begin{cases} \phi_{\rm neut} & (q \leq |q_{\rm c}|) \\ \phi_{\rm neut} - \frac{\beta J}{4q}(q - |q_{\rm c}|)^2 & (q > |q_{\rm c}|). \end{cases} \tag{S26}$$

In general, $\phi_{\rm neut}$, $q_{\rm c}$ and J will depend upon solute size, and whether or not the solute is located in bulk or at the interface.

B. Results

Figures S7, S8 and S9 show $\langle\phi_{\rm solv}\rangle_q$ vs q for $R = 0.240\,{\rm nm}, 0.317\,{\rm nm}$ and $0.415\,{\rm nm}$, respectively, both for the solute in bulk and at the interface. Note that these results have not been corrected for the finite size of the simulation cell: we will correct $\phi_{\rm neut}$ for finite size effects when computing $\Delta_{\rm ads}\psi^{\rm (PLR)}$, where other finite size effects largely cancel.[16] For $R = 0.317\,{\rm nm}$ and $R = 0.415\,{\rm nm}$ we can see that PLR is broadly reasonable for the solute

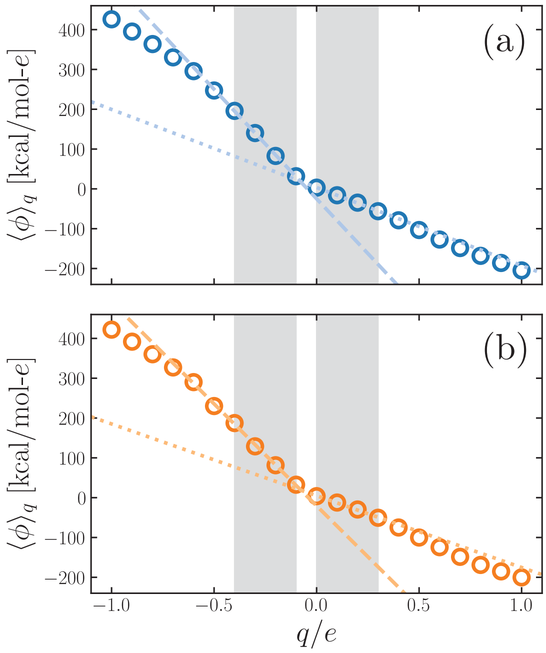

FIG. S7. $\langle \phi_{\text{solv}} \rangle_q$ vs q for $R = 0.240\,\text{nm}$ with the solute located (a) in bulk and (b) at the interface. The dashed and dotted lines show linear fits to the left and right shaded regions, respectively.

in bulk, but some small deviations are seen. These deviations are more pronounced when the solute is at the interface. For $R = 0.240\,\text{nm}$, the above PLR model breaks down at large negative q, but it remains reasonable for smaller values of the absolute charge. By fitting straight lines to the anion and cation response, we can obtain values for q_{c}, $\langle (\delta\phi_{\text{solv}})^2 \rangle_+$ and $\langle (\delta\phi_{\text{solv}})^2 \rangle_-$. The results from using these in Eq. S26 to compute $\Delta_{\text{ads}}\psi^{(\text{PLR})}$ are presented in Fig. 4b in the main article. Results for $R = 0.75\,\text{nm}$ and $R = 1\,\text{nm}$ are not shown because, while anion and cation response do still differ, the degree of nonlinearity is much less on an absolute scale than for the smaller solutes. This makes it challenging to reliably obtain q_{c}.

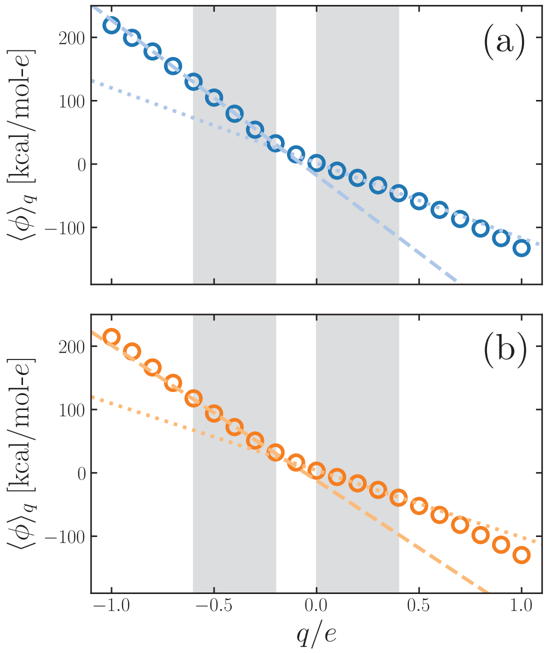

FIG. S8. $\langle\phi_{\mathrm{solv}}\rangle_q$ vs q for $R = 0.317\,\mathrm{nm}$ with the solute located (a) in bulk and (b) at the interface. The dashed and dotted lines show linear fits to the left and right shaded regions, respectively.

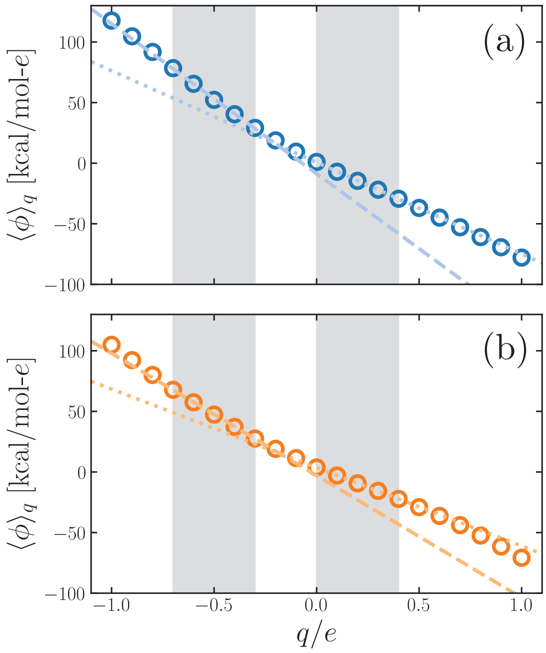

FIG. S9. $\langle\phi_{\mathrm{solv}}\rangle_q$ vs q for $R = 0.415\,\mathrm{nm}$ with the solute located (a) in bulk and (b) at the interface. The dashed and dotted lines show linear fits to the left and right shaded regions, respectively.

IV. CONSTRUCTING $P_0(\phi_{\rm solv})$

In order to compute $F_{\rm chg}(q)$ from Eq. 7, we require $P_0(\phi_{\rm solv})$, the probability distribution of $\phi_{\rm solv}$. For the range of q of interest, i.e. $-1 \leq q/e \leq 1$, sampling P_0 directly (in the absence of solute charge) would yield grossly insufficient data in the extreme wings of the distribution. Instead, we obtain P_0 by histogram reweighting using MBAR.[17] As an illustration, Fig. S10 (a) shows probability distributions $P_q(\phi_{\rm solv})$ of $\phi_{\rm solv}$ at the center of the solute ($R = 0.240\,{\rm nm}$) with different values of q. Using data from simulations across the full range of q, we then construct $P_0(\phi_{\rm solv})$, as shown in Fig. S10 (b).

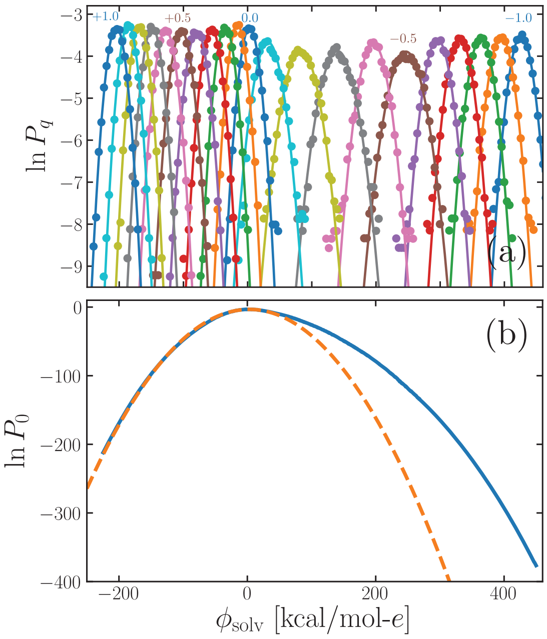

FIG. S10. (a) $P_q(\phi_{\rm solv})$ for $q/e = -1.0, -0.9, \ldots, 0.0, \ldots, 0.9, 1.0$ with $R = 0.240\,{\rm nm}$. Solid lines indicate normalized Gaussian distributions and are included as a guide to the eye. (b) $P_0(\phi_{\rm solv})$ reconstructed from the set of P_q using MBAR[17] (solid line). The dashed line indicates a normalized Gaussian distribution with mean and variance obtained from the simulation at $q/e = 0$. Note that finite size corrections have not been applied to these plots.

REFERENCES

[1]M. A. Kastenholz and P. H. Hünenberger, J. Chem. Phys **124**, 124106 (2006).

[2]For a rigid molecule $\mathbf{\Omega}^{(0)}$ is defined simply by the molecule's orientation, e.g., a set of Euler angles.

[3]P. Hünenberger and M. Reif, *Single-Ion Solvation*, Theoretical and Computational Chemistry Series (The Royal Society of Chemistry, 2011) pp. P001–664.

[4]R. C. Remsing, M. D. Baer, G. K. Schenter, C. J. Mundy, and J. D. Weeks, J. Phys. Chem. Lett. **5**, 2767 (2014).

[5]T. T. Duignan, M. D. Baer, G. K. Schenter, and C. J. Mundy, J. Chem. Phys. **147**, 161716 (2017).

[6]S. M. Kathmann, I.-F. W. Kuo, C. J. Mundy, and G. K. Schenter, J. Phys. Chem. B **115**, 4369 (2011).

[7]R. C. Remsing and J. D. Weeks, J. Stat. Phys. **175**, 743 (2019).

[8]C. C. Doyle, Y. Shi, and T. L. Beck, J. Phys. Chem. B **123**, 3348 (2019).

[9]M. A. Wilson, A. Pohorille, and L. R. Pratt, J. Chem. Phys. **90**, 5211 (1989).

[10]T. L. Beck, Chem. Phys. Lett. **561**, 1 (2013).

[11]A. Arslanargin and T. L. Beck, J. Chem. Phys. **136**, 104503 (2012).

[12]L. Horváth, T. Beu, M. Manghi, and J. Palmeri, J. Chem. Phys. **138**, 154702 (2013).

[13]G. Hummer, L. R. Pratt, and A. E. García, J. Phys. Chem. **100**, 1206 (1996).

[14]R. Lynden-Bell and J. Rasaiah, J. Chem. Phys. **107**, 1981 (1997).

[15]J. P. Bardhan, P. Jungwirth, and L. Makowski, J. Chem. Phys. **137**, 124101 (2012).

[16]S. J. Cox and P. L. Geissler, J. Chem. Phys. **148**, 222823 (2018).

[17]M. R. Shirts, D. L. Mobley, J. D. Chodera, and V. S. Pande, J. Phys. Chem. B **111**, 13052 (2007).
